# Gold nanonetwork film on the ITO surface exhibiting one-dimensional optical properties

**DOI:** 10.1186/1556-276X-7-252

**Published:** 2012-05-15

**Authors:** Akrajas Ali Umar, Iwantono Iwantono, Ariyanto Abdullah, Muhamad Mat Salleh, Munetaka Oyama

**Affiliations:** 1 Institute of Microengineering and Nanoelectronics, Universiti Kebangsaan Malaysia, UKM Bangi, Selangor, 43600, Malaysia; 2 Department of Physics, Faculty of Mathematics and Natural Sciences, Universitas Riau, Pekanbaru, 28131, Indonesia; 3 Department of Material Chemistry, Graduate School of Engineering, Kyoto University, Nishikyo-ku, Kyoto, 615-8520, Japan

**Keywords:** Nanonetworks, Quasi-1D gold nanoparticles, Seed-mediated growth, 1D nanostructures

## Abstract

A network of gold nanostructures exhibiting one-dimensional gold nanostructure properties may become a prospective novel structure for optical, electrical and catalytic applications benefited by its unusual characteristics resulting from the collective properties of individual nanostructures in the network. In this paper, we demonstrate a facile method for the formation of high-density gold nanonetwork film on the substrate surface composed of quasi-1D nanoparticles (typically fusiform) with length *ca*. 10 nm - via reduction of gold ions in the presence of nanoseeds attached surface, binary surfactants of cetyltrimethylammonium bromide and hexamethyleneteramine and Ag^+^ ions. The length of the nanonetworks can be up to *ca*. 100 nm, which corresponds to the aspect ratio of *ca*. 10. The quasi-1D gold nanostructures as well as the nanonetworks were found to be sensitive to the binary surfactants system and the Ag^+^ ions as they can only be formed if all the chemicals are available in the reaction. The nanonetworks exhibit unique 1D optical properties with the presence of transverse and longitudinal surface plasmon resonance absorption. Owing to their peculiar structures that are composed of small quasi-1D nanoparticles, the nanonetworks may produce unusual optical and catalytic properties, which are potentially used in surface-enhanced Raman scattering, catalysis and optical and non-linear optical applications.

## Background

One-dimensional gold nanostructures, such as nanorods, nanowires, etc., have continued to be the objective of the research in the controlled-shape synthesis nowadays due to its notable catalytic, electrical, magnetic and optical characteristics [1-4]. Their optical absorption properties, in particular, are characterised by the presence of two unique strong localised-surface plasmon resonance [5] bands near the 500 (transverse mode) and 650 nm (longitudinal mode) but tunable within the region of *ca*. 400 to 550 nm and of *ca*. 600 to 800 nm, respectively. This enables them to produce peculiar optical properties for use in a broad range of applications including nanophotonics [6-11], light-emitting diode [12], photovoltaics [13-17] and photosynthesis efficiency enhancement [18,19] and optical sensing applications [20]. For example, nanorods demonstrate a unique profile and enhanced energy of electron-wave scattering upon exposure to the electromagnetic irradiation resulting from their anisotropic localised-plasmonic excitation character [21,22], placing them as a potential agent for photodynamic therapy of cancer cells [19,23-26], optical contrast agent and for diagnostic imaging applications [20,27,28]. As gold itself has high compatibility to a wide range of biomolecules and its peculiar surface morphology is bounded normally by highly-energetic planes ({110} and {100}) [1,3,29-31], these nanostructures become an attractive candidates for catalysts in bio-organic reactions [32,33] and chemical sensing applications [20,23].

A wide range of methods are currently available for the preparation of gold nanorods with controlled shape, yield and aspect ratios in the solution. The synthetic procedure as well as the mechanism of the formation has also been well-summarised in many recent reports [1,3,5,29]. To mention a few examples, cetyltrimethylammonium bromide (CTAB) surfactant-assisted seed growth method by Murphy et al. appeared as a versatile approach for the synthesis of gold nanorods with a controlled aspect ratio in the solution [5,29,34]. A much improved technique was later demonstrated by El-Sayed and co-workers, in which, by modifying the seeding and adding of Ag^+^ into the growth solutions that was used by Murphy, nanorods with a yield of nearly 100 % could be achieved [35]. Effective facet-selective gold deposition process in the presence of silver ions in the growth solution was considered as the key driving factor for the high-yield gold nanorod formation. However, in contrast to such outstanding achievement in solution phase, their growth directly on the surface has not yet been developed. Since many applications, such as optoelectronic, catalyst and surface-enhanced Raman scattering (SERS), required that they are attached onto the surface, the technique for growing the nanorods directly on the surface should be developed. It is true that the solution phase prepared gold nanorods may be transferred onto the surface via, for example, Langmuir-Blodgett [36] or drop and spin casting assemblies [37]; however, they are likely intricate and inadvertently expose the nanostructures to possible properties disruption during the process. Therefore, to develop a method that enables the shape-controlled growth directly on the surface is crucial.

Recently, we have grown metal nanoparticles directly on the surface using a seed-mediated growth method [38-40]. Gold nanoparticles with a variety of shapes, such as nanoplates, nanocubes, etc., have been successfully prepared directly on the surface [41,42]. As for 1D morphology, particularly in nanorods, considerable efforts have been devoted by us to synthesise them directly on the surface. Unfortunately, so far their yield was relatively low so that the unique 1D optical properties that are indicated by the presence of two separate bands, i.e. transverse and longitudinal SPRs, could not be detected on their optical absorption spectra. Actually, we have also attempted many approaches to improve the yield including the use of the well-known growth solutions for gold nanorods such as prescribed by Murphy's [3,29,34,43], El-Sayed's [35] and Korgel's [44] groups combined with our method. Unfortunately, again, these attempts failed. This could be the result of unique growth characteristic of gold nanostructures on the surface, which might be influenced by many factors including substrate surface-nanoseeds interaction effect, substrate surface-solution interface characteristics (e.g. wet-ability), and the nature of the precursors transport to the growing crystallite on the surface, which produce a different crystal growth compared to that of in the solution. We note that the formation of 1D gold nanoparticles directly on the surface is a remarkably challenging task, however, since the anisotropic effect of the nanostructure in the applications are very special and even superior in many aspects over other morphologies, the synthesis of gold nanoparticles with alternative morphology directly on the surface but producing properties that exhibit those in one-dimensional nanostructures is of particular interest.

Here, we report the synthesis of high-density networked gold nanostructures directly on the surface exhibiting unique one-dimensional gold nanoparticle characters. By combining a special effect of AgNO_3_ in the formation of gold nanorods to our new growth solution that contains cetyltrimethylammonium bromide and hexamethylenetetramine using a seed-mediated growth method, high-yield networked gold nanostructures can be realised directly on the surface. Optical absorption spectrum analysis on the sample surprisingly revealed that the nanostructures exhibit one-dimensional properties, reflected by the presence of two plasmonic bands on their spectrum. Therefore, they could become a replica of gold nanorods on the surface to be used in SERS, catalysis and plasmonics enhanced optoelectronics devices.

## Methods

**Figure 1:**
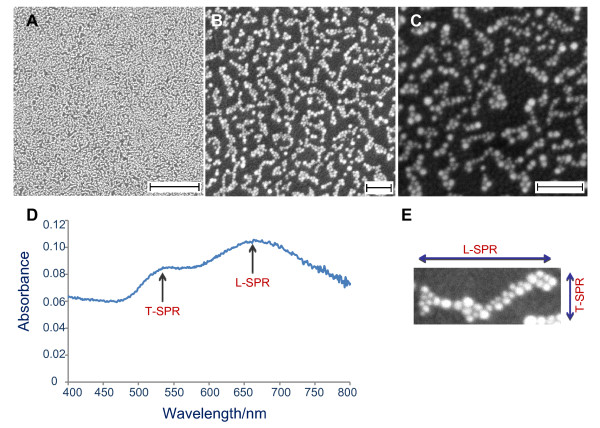
**FESEM images and high magnification of networked gold nanoparticles and optical absorption spectra of gold nanostructures.** (**A** to **C**) FESEM images of networked gold nanoparticles grown on ITO surface for different magnification**.** The sample was prepared using a growth solution that contains 0.5 mL of 0.01 M HAuCl_4_, 12 mL of 0.1 M CTAB, 8 mL of 0.1 M HMT and 0.1 mL of 0.1 M ascorbic acid. (**D**) Optical absorption spectra of gold nanostructures showing two plasmonic characters, namely transverse (*ca*. 525 nm) and longitudinal (*ca*. 625 nm) surface plasmon resonance. (**E**) Shows high magnification of networked gold nanoparticles and possible origin of LSPR on the nanostructures. Scale bars are 1 μm in A and 100 nm in B to C.

Networked gold nanoparticles were grown directly on the surface via our seed-mediated growth method. This technique comprises a two-step process, namely seeding and growth processes. In typical procedure, the seeding process was carried out via *in situ* reduction of gold salt on the substrate sample [45]. Briefly, a clean ITO substrate (surface resistance *ca*. 30 Ω/sq, CBC Ings Co. Ltd., Tokyo, Japan) was immersed into a solution that contains 0.25 mM HAuCl_4_ and 0.25 mM trisodium citrate. The substrate was kept undisturbed in the solution at 28°C for 1 h. After that, 0.5 mL of 0.1 M ice-cooled NaBH_4_ was added to the solution, and immediately, the gold nanoseeds formed in the solution as well as on the surface. The sample was then kept in the solution for another hour. After that, the sample was removed, rinsed with pure water and dried with a flow of nitrogen gas. Using this approach, high-density gold nanoseeds will be formed on the surface (see Figure 1A).

The growth process was carried out by immersing the substrate that has been treated with the nanoseeds into a growth solution that contains 0.5 mL of 0.01 M of HAuCl_4_, 8 mL of 0.1 M CTAB, 12 mL of 0.1 M hexamethylenetetramine, 0.3 mL of 0.1 M ascorbic acid and 40 μL of 0.01 M of AgNO_3_. The sample was kept undisturbed for 4 h at 28°C for the growth process. If this condition is used, the final concentration of each reagent is 0.25, 40, 60 and 1.5 mM and 20 μM for HAuCl_4_, CTAB, hexamethyleneteramine (HMT), ascorbic acid and AgNO_3_, respectively. Finally, the sample was removed, rinsed with plenty of pure water and dried with a flow of nitrogen gas. The growth solution used in the present study was a new growth solution developed by our group of which was the modification of our standard solution for preparing gold nanotripod in solution [46] as our initial expectation to also realise the formation of nanotripods on the surface if using similar growth solution. There are several modifications that have been made to the standard solution, namely the AgNO_3_ was new here and the NaOH is not required for the present study as it was in the preparation of gold nanotripod.

The morphology of the gold nanostructures grown on ITO surfaces was characterised using a field-emission scanning electron microscopy (FESEM) (JSM-7400 F, JEOL Ltd., Akishima, Tokyo, Japan). The optical absorption spectrum of the samples was obtained using Perkin Elmer Lambda 900 UV/VIS/NIR spectrometer (Waltham, MA, USA).

## Results and discussion

We noted again here that the growth solution used in this project was actually a modification to the solution that was originally used to prepare gold nanotripods in solution phase [46]. Prior to modifying the solution, we actually have used such original solution to grow the attached-nanoseeds on the surface via a seed-mediated growth method. Actually, we expected that similar morphology, i.e. nanotripods, would be realised on the surface. Unfortunately, neither nanotripods nor nanorods were obtained but instead spherical gold nanoparticles were formed, reflecting unusual heterogenous gold deposition on the nanoseed surface emerged as the results of the surface effect. Thus, shape-controlled growth on the surface often yields limited success.

The original growth solution for the nanotripods contained two special surfactants, namely CTAB and HMT. As an attempt for obtaining 1D gold nanoparticle growth from the nanoseeds on the surface and being inspired by the fact that the AgNO_3_ promotes the formation of nanorods in solution [35], we added a minute amount of AgNO_3_ into the original solution. After being immersed for 4 h in the growth solution, a purple-blue colour was formed on the surface. Normally, this kind of colour only appears on the gold nanostructures with one-dimensional morphology, such as nanorods or nanowires [3,29,35]. Therefore, this result signifies that one-dimensional morphology of gold nanoparticles might have formed on the surface. Optical absorption spectrum collected from the samples surprisingly showed the presence of two plasmonic bands at 520 and 680 nm. In agreement with the observed purple-blue colour, two-banded absorption spectrum is also an indication of the formation of one-dimensional gold nanostructures on the surface. We then carried out a FESEM characterisation on the as-prepared samples. To our surprise, only high density networked string-like structures of gold nanoparticles were formed on the surface covering the entirety of the substrate surface instead of nanorods or other 1D morphologies, to which such unique two plasmonic characters can be related to. The optical absorption and the FESEM results are shown in Figure 1. However, as revealed in the high-resolution FESEM image of the samples (see Figure 1C), actually the networked structures were mostly composed of quasi-1D nanoparticles (e.g. fusiform) with size (length) approximately 10 nm that aligned side-by-side with each other. The length of the nanonetworks can be up to *ca*. 100 nm, which corresponds to the aspect ratio of *ca*. 10. Despite the fact that the spherical nanoparticles also formed the networked structures, the network is normally relatively shorter than those composed of the quasi-1D nanoparticles. Even in most cases, they seemed to be an aggregate instead of a long-range networked morphology. However, no nanorods, nanowires or other 1D nanostructures were observed on the image, confirming that one-dimensional optical properties are solely produced by the networked-gold nanoparticles on the surface. As revealed in the image, the quasi-1D structures that composed the networked structure are considerably small with average length and diameter in the range of *ca*. 2 to3 nm and 10 to 15 nm, correspondingly. Such small 1D nanostructures might have prospective use in catalysis and sensing applications such as the possibility of producing peculiar properties as the results of anisotropic morphology and quantum effect.

As evident in Figure 1A,B,C, no perfect nanorods or nanowires were obtained on the surface. Therefore, the presence of one-dimensional optical properties could be directly related to the networked structure (nanonetworks) that formed linear chains on the surface. Thus, the transverse surface plasmon band, the shorter wavelength band, can be easily attributed to the oscillation of the free electron system toward the short axis of the nanonetworks. Meanwhile, the longitudinal plasmonic band, i.e. the longer band, was related to a long-range plasmonic coupling [47] amongst the nanoparticles in the networked structure (see Figure 1E). Actually, the appearance of a longitudinal plasmonic band from the networked nanoparticles here is quite unusual and is predicted as resulting from the unique 1D morphology of the individual nanoparticles in the nanonetworks. According to our earlier results [38-40], in most cases, such unique 1D plasmonic characteristics were not observable if the network was composed of spherical nanoparticles. Therefore, the appearance of LSPR in these nanonetworks in the present study indicates a peculiar dependence of long-range plasmonic coupling on shape at this length-scale regime [47]. Thus, more unusual properties will then be expected to arise by the quasi-1D but assembled in a network structure.

**Figure 2:**
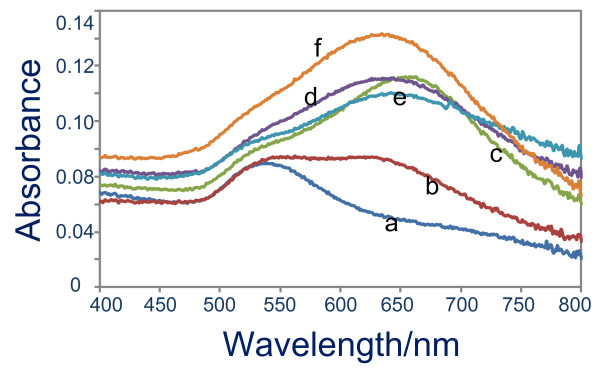
**Optical absorption spectra.** Gold nanostructures on the surface prepared using several Ag^+^ concentrations, namely (**a**) 0, (**b**) 10, (**c**) 20, (**d**) 30, (**e**) 40 and (**f**) 50 μM. Other reagents were kept unchanged as that in Figure 1. Growth time was 4 h.

**Figure 3:**
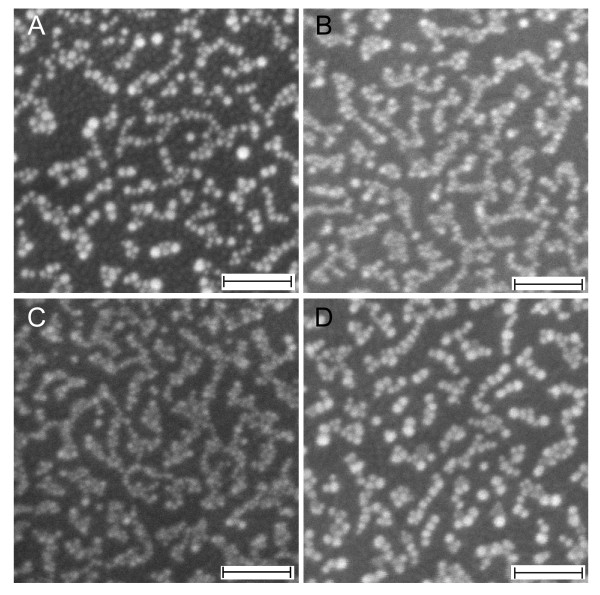
**FESEM images.** Selected gold nanostructures' growth prepared using several AgNO_3_ concentrations, of which their corresponding absorption spectra are shown in Figure 2. (**A**) 10, (**B**) 20, (**C**) 30 and (**D**) 50 μM. Scale bars are 100 nm.

As has been noted earlier, the AgNO_3_ might have played a substantial role behind the formation of quasi-1D gold nanoparticles, which is the basis for the nanonetwork structure. To obtain a detailed understanding on its role in this process, we examined the growth characteristic of gold nanostructures on the surface in the presence of several AgNO_3_ concentrations, namely 10, 20, 30, 40 and 50 μM, via UV-vis absorption spectroscopy and FESEM imaging. Figure 2 is the related optical absorption spectra of the samples. As judged from the curve a, neither quasi-1D nor nanonetworks were formed when the AgNO_3_ was absent in the reaction. However, 1D gold nanostructures might have effectively produced when the AgNO_3_ as low as 10 μM was used as the appearance of two plasmonics bands at *ca*. 520 (TSPR) and 630 nm (LSPR) (see curve b). The quasi-1D nanostructures as well as nanonetworks formation might be optimum when the AgNO_3_ concentration used was 40 μM. It is indicated by a maximum shifting of the LSPR to red, reflecting the improvement of the aspect ratio of the nanonetworks (curve c). If the AgNO_3_ concentration was higher or lower from this value, lower LSPR peak position was obtained, suggesting a decrease in the aspect ratio of the nanonetworks. The FESEM results as shown in Figure 3 further verified such growth characteristic of the nanostructures on the surface under the present treatment. For example, effective nanonetworks formation was confirmed when AgNO_3_ concentration of as low as 10 μM was used in the reaction (see Figure 3A). This is probably the origin of the observed LSPR band in the spectra, formed via the presence of a long-range plasmonic coupling process in the nanonetworks. As has been noted earlier, spherical nanoparticle chains did not produce such optical properties resulting from a long-range plasmonic coupling amongst the nanoparticles in the chain. Thus, special nanostructure morphology, i.e. quasi-1D, should be present in the nanonetworks in order to support effective long-range plasmonic coupling via optimum edge-to-edge quasi-1D arrangement. The available FESEM images actually did confirm the nanostructures that composed the nanonetworks are quasi-1D morphology instead of spherical nanoparticles. It is true that high-resolution FESEM images were not available to support the claim. However, the present optical absorption spectra results that show 1D optical property, produced from effective long-range plasmonic coupling, are strong evident for the formation of such nanostructures morphology in the nanonetworks. However, a complete analysis on the detailed morphology of the individual nanostructures on the nanonetworks including high-resolution electron microscopy is being pursued and will be reported in a different publication. Large-scale nanonetworks were effectively formed covering the entirety of the surface when optimum AgNO_3_ concentration was used, namely 20 μM (see Figure 3B). One point to be noted here is that the actual length of the nanonetworks obtained using this condition was relatively the same with that shown in Figure 3A; however, their shorter axis was relatively smaller. Thus, the aspect ratio of the nanonetworks increased, shifting the LSPR to red. As has been earlier probed in the absorption spectra, further increase in the AgNO_3_ added decreased the aspect ratio of the nanonetworks. Judging from the FESEM results, we pointed out the following facts as the reason for the process: The density of the nanonetworks increased with the increasing of AgNO_3_ concentration. As revealed in Figure 3C, despite the fact that the short axis of the nanonetworks decrease due to the increase in the density and possible strong coupling along the short axis of the nanonetworks, further red-shifting in the LSPR was not achieved but blue-shifting instead. A different structure was obtained when high concentration of AgNO_3_ was added, namely 50 μM. As can be seen from the Figure 3D, nanonetworks that are composed of relatively bigger spherical nanoparticles were typical of the nanostructures product. The increase in the individual nanoparticle size might be due to a kind of ‘steric’ hindrance of surfactant adhesion onto the nanoparticles surface in the presence of high concentration of AgNO_3_, accelerating the growth of individual nanoparticles.

**Figure 4:**
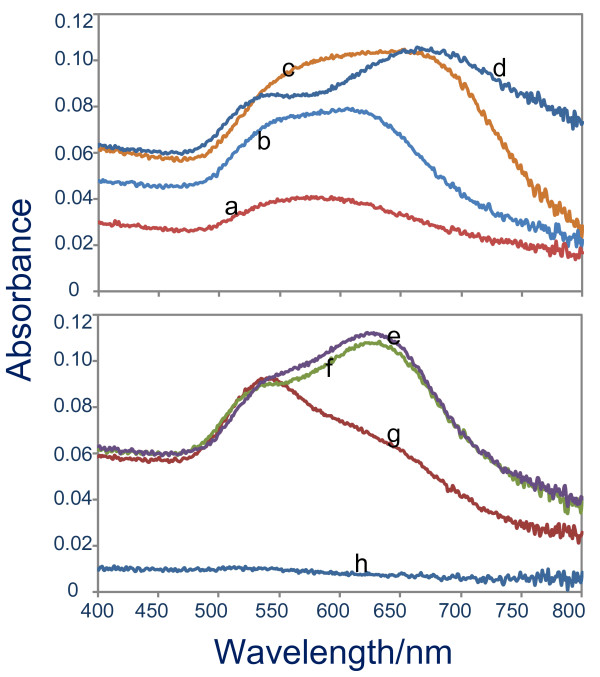
**Optical absorption spectra.** Gold nanostructures on the surface prepared using optimum AgNO_3_ concentration namely 20 μM, with several CTAB to HMT ratio (millilitre to millilitre)**.** (**a**) 20:0, (**b**) 18:2, (**c**) 15:5, (**d**) 12:8, (**e**) 10:10, (**f**) 8:12, (**g**) 5:15 and (**h**) 0:20. Other chemicals were unchanged, namely 0.5 mL of 0.01 M and 0.1 mL of 0.1 M for HAuCl_4_ and ascorbic acid, respectively.

**Figure 5:**
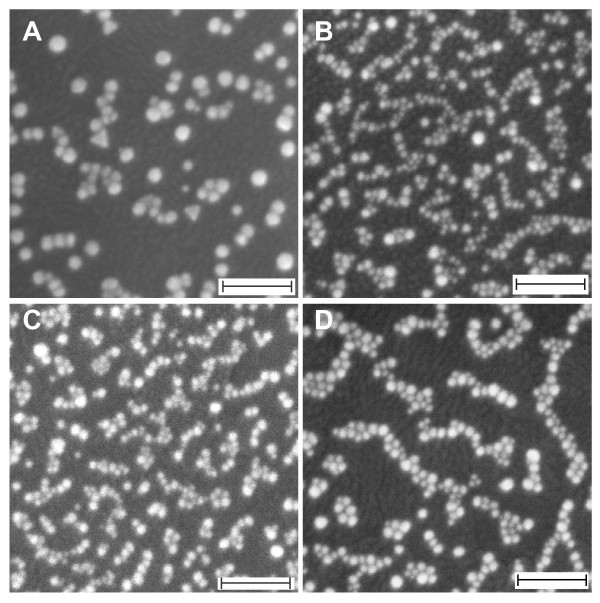
**FESEM images.** Gold nanostructures prepared using selected CTAB to HMT ratio (millilitre to millilitre), of which their corresponding absorption spectra are shown in Figure 4. (**A**) 20:0, (**B**) 12:8, (**C**) 10:10 and (**D**) 8:12 mL. Scale bars are 100 nm.

While AgNO_3_ drives the formation of 1D crystal growth of the nanoseeds, both CTAB and HMT via their combinative effect are also crucial in this process. In a typical case, no 1D optical characteristic was obtained if one of the surfactants was absent in the reaction. Typical optical absorption spectra of gold nanostructures prepared using several concentration ratios of CTAB and HMT (millilitre to millilitre ratio) are shown in Figure 4. As revealed in the spectra, both surfactants must be present in the reaction to give an optimum 1D optical characteristic, which is indicated by the longest LSPR peak position when CTAB to HMT ratio is 12:8 (see curve d). By keeping the cumulative concentration of both surfactants unchanged, if the ratio was increased or decreased, the LSPR peak position was blue-shifted compared to the optimum one, a sign of a decrease in the aspect ratio of the 1D nanostructures. It needs to be noted that limited number of gold nanoparticles probably grew on the surface when only surfactant HMT presented in the reaction, as judged by its low optical absorbance (see curve h). This could be due to a weak capping nature of the HMT to Au^+^ so that the Au ^+^ reduction occurred in the solution instead on the nanoseeds surface. Selected FESEM image of the samples as shown in Figure 5 further confirms and provides detailed pictures of gold nanostructures' growth characteristic on the surface under several CTAB to HMT concentration ratios. For example, high-yield nanonetworks were obtained when the CTAB to HMT ratio of 12:8 was used. In good agreement with the optical absorption spectrum as shown in Figure 4, the yield was found to decrease when the ratio was increased (see Figure 5C for CTAB to HMT ratio of 10:10). Bigger-sized nanoparticle network was even obtained if the ratio further decrease to 8:12. On the basis of these results, it can be worthwhile concluding that combinative function of surfactants plays a strategic role in the formation of quasi-1D morphology and then the nanonetwork structures on the surface.

It needs to be noted here that the individual nanoparticles which compose the nanonetworks were actually mainly the quasi-1D gold nanostructures formed by a unique interplay amongst the reagents under the present condition. We hypothesised that a facet-selective surfactant adhesion, which is driven by the Ag^+^ via its unique underpotential deposition nature onto the nanoseed surface, could be the main factor for the formation of the structures. However, the exact mechanism is not clear at the moment, especially the nature of the surfactants adhesion as well as the interplay amongst them and the Ag^+^. Although the following facts could be considered: (a) Quasi-1D nanostructures were not formed when one of the surfactants was absent. The quasi-1D nanoparticles may be formed at any concentration ratio between the two surfactants but optimum at the CTAB to HMT ratio of 12:8. This reveals that the combinative function of surfactant here is crucial. (b) The quasi-1D nanostructures were also not formed when Ag^+^ is absent in the reaction. In fact, a diluted Ag^+^ concentration (*ca*. 10 μM) projected the quasi-1D nanoparticles growth and optimum at a concentration of approximately 20 μM. These indicated that the interplay amongst the surfactants and the Ag^+^ is necessary for the formation of quasi-1D nanoparticles. It is widely known that the Ag^+^ may adsorb onto the gold nanoseed surface and then induces a one-dimensional crystal growth in the nanoseeds, certainly in the presence of surfactant. This scheme might probably also be valid to the present condition. However, since the system was on the solid surface, the growth characteristic should be different. It is true that the bromide ions were recognised to have a key effect in the promotion of nanorod morphology growth in the solution phase via effective adsorption onto the lower-energy surface of the nanostructures, i.e. (111) and (100), and play as a steric hindrance on these planes, which lead to a nanorod shape formation with growth direction toward (110) [48]. However, since in this experiment the quasi-1D morphology were not formed in the absence of Ag ion, the role of Br ion in the formation of quasi-1D nanostructures on the surface is not relevant. It is also true that the AgBr complexes may also be formed during this process. Since these complexes are relatively inactive compared to the Br ions as well as their quantity might be considerably small as the result of effective Ag ion deposition onto the Au nanoseed via effective underpotential deposition process, this chemical was believed to have minor effect on the promotion of quasi-1D gold nanostructures.

**Figure 6:**
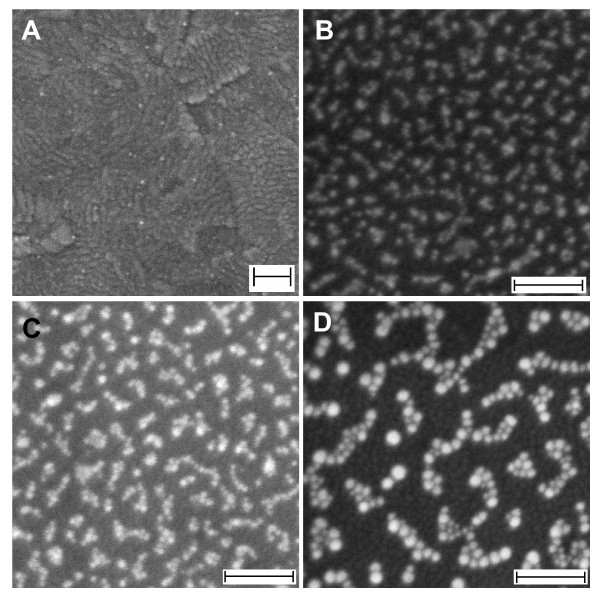
**FESEM image of gold nanostructures.** Nanostructures grown for (**A**) 0 min (nanoseeds), (**B**) 30 min., (**C**) 1 h and (**D**) 4 h, using the optimum growth solution as that in Figure 1. Scale bars are 100 nm.

Meanwhile, on the network structures, their formation is assumed as an attempt to minimalise the high surface energy of the quasi-1D nanostructures that formed under the present condition. Actually, the initial nanoseeds on the surface were randomly distributed without any specific orientation (see Figure 6A). After being grown in the growth solution, adjacent nanoseeds grew into a quasi-1D morphology and aligned with each other forming the nanonetwork structures (see Figure 6B,C). Meanwhile, the nanoseeds that were far from the nanonetworks may have grown into bigger spherical nanoparticles or simply dissolved into the bulk solution via the Oswald annealing process and then supported the growth of bigger nanostructures on the surface. As revealed in the FESEM images, the nanonetwork structures have been observed on the sample that was grown for 30 min. In spite of that fact, their formation may be earlier. The formation of these structures via aggregation amongst the nanostructures is not applicable in this process. It is because the nanostructures were grown from the nanoseeds attached to the surface so that the possibility of the nanostructures migration on the surface could be minimalised. The aggregation of the nanostructures from the bulk solution and then attached onto the surface is also irrelevant here since the mature nanostructures are only on the nanoseeds that are attached to the surface.

## Conclusions

We have demonstrated a facile formation of gold nanonetworks from the attached nanoseeds on the surface that exhibit a 1D structure property via a combinative effect of CTAB and HMT surfactants and Ag^+^ in the growth solution. In a typical case, the nanonetworks were mainly composed of small quasi-1D gold nanostructures that probably aligned due to surface energy minimalisation. The quasi-1D nanoparticles as well as the nanonetworks were very sensitive to the CTAB, HMT and Ag^+^ in the reaction and neither quasi-1D nanostructures nor nanonetworks were formed if one of the three chemicals was absent. At this stage, the dimension control of both the quasi-1D structures and the nanonetworks, in particular the aspect ratio, was not yet achieved. An intensified further attempt to achieve that control is underway. Owing to its unique structure that composed of small quasi-1D gold nanostructures, the nanonetworks may produce unusual optical and catalytic properties that make them highly demanded in photonic, catalysis and surface-enhanced Raman scattering applications.

## Competing interests

The authors declare that they have no competing interests.

## Authors' contributions

AA carried out the nanostructure preparation and characterisation. AAU designed the concept and experiment, analysed the results and drafted, revised and finalised the manuscript. II participated in the data analysis. MMS provided the facilities and discussed the results. MO provided the concept of the growth process of the nanostructures. All the authors contributed to the preparation and revision of the manuscript. All authors read and approved the final manuscript.
